# Continuous Vital Monitoring During Sleep and Light Activity Using Carbon-Black Elastomer Sensors

**DOI:** 10.3390/s20061583

**Published:** 2020-03-12

**Authors:** Titus Jayarathna, Gaetano D. Gargiulo, Paul P. Breen

**Affiliations:** 1 The MARCS Institute, Western Sydney University, Milperra, NSW 2560, Australia; P.Breen@westernsydney.edu.au; 2 School of Engineering, Western Sydney University, Penrith, NSW 2751, Australia; G.Gargiulo@westernsydney.edu.au; 3 Translational Health Research Institute, Western Sydney University, Campbelltown, NSW 2560, Australia

**Keywords:** vital parameter monitoring, sleep monitoring, conductive polymer, home sleep test, continuous respiratory monitoring

## Abstract

The comfortable, continuous monitoring of vital parameters is still a challenge. The long-term measurement of respiration and cardiovascular signals is required to diagnose cardiovascular and respiratory diseases. Similarly, sleep quality assessment and the recovery period following acute treatments require long-term vital parameter datalogging. To address these requirements, we have developed “VitalCore”, a wearable continuous vital parameter monitoring device in the form of a T-shirt targeting the uninterrupted monitoring of respiration, pulse, and actigraphy. VitalCore uses polymer-based stretchable resistive bands as the primary sensor to capture breathing and pulse patterns from chest expansion. The carbon black-impregnated polymer is implemented in a U-shaped configuration and attached to the T-shirt with “interfacing” material along with the accompanying electronics. In this paper, VitalCore is bench tested and compared to gold standard respiration and pulse measurements to verify its functionality and further to assess the quality of data captured during sleep and during light exercise (walking). We show that these polymer-based sensors could identify respiratory peaks with a sensitivity of 99.44%, precision of 96.23%, and false-negative rate of 0.557% during sleep. We also show that this T-shirt configuration allows the wearer to sleep in all sleeping positions with a negligible difference of data quality. The device was also able to capture breathing during gait with 88.9–100% accuracy in respiratory peak detection.

## 1. Introduction

The human body can be described as a continuously running machine with electrical activity and mechanical movement. Many vital signs, as we measure them, are a byproduct of these dynamics and are used to indicate the general health of the individual through comparison with known healthy values. The three most basic vital signs are respiration, heartbeat, and body temperature, which can be sensed directly by another human without any medical equipment. Respiration is observed as rhythmic chest/abdomen movement or sensing the air moving in/out of the nostrils or mouth. The heartbeat can be observed either by capturing the electrical signal within the heart, its direct mechanical response, or the subsequent pulse at the arm, neck, or groin. Body temperature cannot be specifically measured without a thermometer; however, a healthy human can touch and determine when a body is abnormally hot or cold. All three vital signals have established acceptable ranges indicating when an individual is generally healthy or unhealthy. Among the three vital signs, both respiration and heartbeat may be measured by quantifying mechanical movement.

A healthy, independent human does not need continuous vital sign monitoring except perhaps to evaluate their physical performance. If an individual is truly ill, they will undoubtedly be connected to a myriad of available medical devices to monitor a large variety of physiological data. However, it is often the case that no continuous physiological monitoring is maintained following the acute phase of treatment and recovery. There are several reasons why this may be the case, including the cost of additional equipment and the benefit of the patient not being tethered to equipment so they can ambulate with minimal risk. However, as the cost of healthcare continues to rise, there is increasing interest in minimizing avoidable inpatient hospitalizations. A light-touch physiological monitoring system can aid in enabling this need. In the first instance, it could provide the clinician with accurate data that demonstrates that the patient is sufficiently stable to be discharged. A connected device could potentially go with the patient, again providing assurance that their health is maintained during and following transition to their home or care provider. This may have an additional benefit of identifying risk factors that could lead to readmission, allowing earlier preventative intervention. To our knowledge, there is no existing device developed with this application in mind. Such a device should fulfill the following four basic requirements.

1. Low cost.
2. Simple setup procedure.
3. Comfortable and suitable for multiple day/night use.
4. Accurate representation of the physiological metrics of interest.

These four requirements are not mutually exclusive, and the increased fulfillment of one requirement can easily degrade the fulfillment of another. The success of such a device depends on how the designers tune the four parameters such that it can provide the best possible solution. The simplicity, “invisibility”, and comfort of the designed device depend on the materials and monitoring methods employed. For example, measuring respiration using tubes inserted into nostrils, face masks, or acoustic sensors mounted on the nose or throat could provide accurate measurements but would not satisfy the requirement regarding user comfort [1,2]. Completely noninvasive methods such as video [3,4,5], vibration, or Doppler effects [6,7] are comfortable to users but may not be simple to set up or provide the required continuous accuracy. In our previous work, we have observed that polymer-based conductive rubber or conductive stretchable fabric when used as displacement and vibration sensors could introduce a balanced middle ground between these two approaches [8,9,10,11,12,13]. These ‘morphic’ sensors are worn tight to the body, but not with any significant compressive force; furthermore, they are unobtrusive and are comfortably worn.

Table 1 shows a summary of the currently available wearable solutions for vital parameter monitoring. The table is divided into commercially available products and research or prototype-only devices. The intended purpose or use of each device is listed. The capability of capturing heart rate (HR) and respiratory rate (RR) are indicated, along with the corresponding technologies used to achieve these measurements.

One noticeable pattern of the commercial products (shaded gray on Table 1) is the lack of respiratory monitoring. Among these, only Hexoskin is capable of providing continuous respiratory monitoring using respiratory inductance plethysmography (RIP) bands. RIP involves the AC polarization of a conductive wire (usually a rubber-shielded copper wire) and measuring the inductance change [23]. The RIP bands provide excellent reproducibility allowing respiratory volume measurement. However, it also involves more complex instrumentation without advantages in signal to noise ratio (SNR) compared with a DC polarized resistive band approach. Mutual inductance between RIP sensors is another issue that either requires multiple high-Q oscillators with sufficient frequency separation or a time-division multiplexing scheme.

Everion claims that it can provide RR; however, we did not find any evidence that the measures are accurate enough to be useful. Huwiler states, “Unfortunately, the data quality of the Everion measurement of the respiratory rate was low, and therefore, could not be included into the analysis” in a sleep monitoring experiment setup [24].

Apart from the T-shirt-like wearable solutions, there are several specialized devices in the market to monitor vital signals during sleep. These at-home sleep monitoring devices are designed to aid the diagnosis of sleep disordered breathing, primarily obstructive sleep apnea [25]. Apnealink air [26], Nox T3 [27], Philips Alice PDx [28], and Alice NightOne [29] are some of the more popular devices in use. These devices support multiple channels such as respiratory monitoring using RIP bands or nasal pressure cannula, heart rate monitoring using electrocardiogram (ECG) or peaks of finger measurement (PPG) and blood oxygen saturation monitoring. The major disadvantages of these devices are their bulkiness and user comfort. The main controller units of Apnealink air (6.2 cm × 10.2 cm), Nox T3 (7.9 cm × 6.3 cm), Alice PDx (12.7 cm × 7.62 cm), and Alice NightOne (10.34 cm × 6.78 cm) are relatively large devices that need to be attached on the chest during sleep. The rigid structure and numerous sensing apparatus attachments do not allow the user to sleep in all sleep positions comfortably.

Research-based prototype devices and experiments typically feature technical methods that are not yet easily realizable in mass produced products. Wireless antenna signal attenuation-based respiratory monitoring produces a very low SNR signal, while flexible fiber Bragg grating involves complex instrumentation that is not suitable for low-power, smaller “invisibles”. The Phyjama [22] is an interesting approach to measure respiration and pulse using layered fabric sensors. The fabric sensors are stitched to a T-shirt as square pads on multiple locations and the pads change resistance with pressure applied to each pad. However, it is “designed for close contact with the skin and unsuitable for loosely worn clothing”. The patches rely only on passive pressure applied to the fabric (such as during sleep, where at least one patch is compressed against the body), so the device is unusable in sitting and standing positions due to the lack of passive pressure against the T-shirt. In our experience with polymer-based resistive elements or conductive fabric [30,31,32], resistive change has a non-linear relationship where the highest sensitivity is obtained within the low strain region, <2% stretch. Therefore, when conductive fabric or carbon black elastomers are used, the sensors are not particularly tight in use, requiring less compressive force either from the T-shirt material or substrate layer to follow the body movement closely and accurately. 

This paper focuses on exploring the use of these ‘morphic’ sensors to extract mechanical movements as a means of monitoring respiratory and cardiac function. As the intended use case is limited to the context of a patient following acute care and in/following the transition to the home/care provider, monitoring requirements are limited to low-level activity (walking) and in bed (sleep). The first part of this paper describes the rationale and technical development of the morphic sensor-embedded T-shirt. This is followed by bench-testing and use-case matched proof-of-concept experiments.

## 2. Material and Methods

This paper evaluates the performance of flexible electroresistive band (ERB) sensors to monitor mechanical physiological body movements. These movements are essentially regional body volume changes transferred to conductive/electroresistive materials embedded in flexible clothing. There are a number of varieties of conductive polymers/fabrics available in the current market; however, the majority of these materials are not available commercially and are still in the research stage. Stretchable electroresistive sensors incorporate two main components, the conductive component (e.g., carbon black, graphene, nanowires, or metal elements such as silver, gold, nickel, and copper) with flexible support material (e.g., silicon-based elastomers, rubber-based elastomers) [30]. Graphene-based material shows better dynamic characteristics and performance but is in the early stage of research with limited availability. Conductive polymers based on carbon black and metal-doped fabrics are widely available due to their widespread use for electromagnetic interference shielding applications.

### 2.1. Sensors

**Figure 1:**
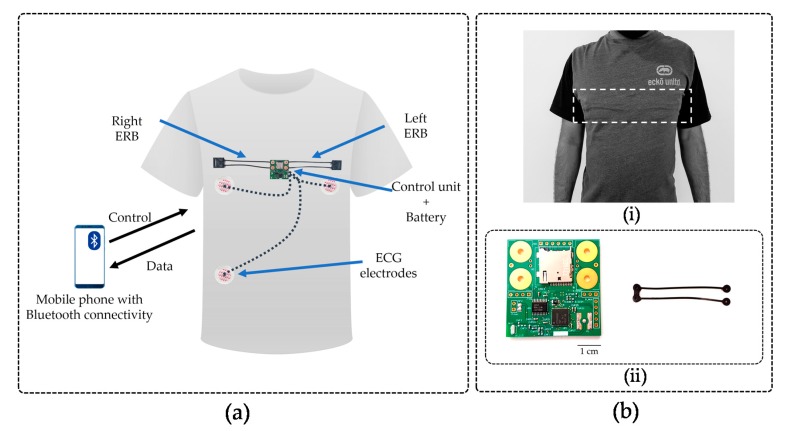
(**a**) Abstract view of the system design highlighting the location of the printed circuit board (PCB), U-shaped sensor band, and ECG electrodes. (**bi**) External view of VitalCore prototype when electronics and sensors are attached to the rear side of the fabric using interfacing. The dashed box highlights the region where the electronics are placed. (**bii**) Top side of the PCB and the U-shaped carbon black conductive rubber sensor. The PCB shows the microcontroller unit (MCU), Bluetooth antenna, analogue to digital converter (ADC), secure digital (SD) cardholder and four placeholders to connect two electroresistive bands (ERBs).

For this work, we have manufactured a bespoke U-shaped carbon black rubber sensor (Figure 1(bii)). This shape allows for a single point electrical connection to the main circuit removing the need for a return wire. The electrical parameters of the sensor are reported in Table 2. It is important to note that the resistance change with length is not perfectly linear. In addition, the rubber shows an exponentially decaying voltage under constant current before stabilizing.

While we are primarily interested in monitoring vital signals (cardiac and respiratory function) using flexible conductive materials, we also incorporated accelerometry to capture the body position and activity and investigate the impact of movement artefacts. An electrocardiogram (ECG) frontend is also included as a cardiac activity reference for comparison.

### 2.2. Hardware and Electronics

The hardware platform is designed such that it can be integrated into a T-shirt allowing maximum “invisibility”. Data need to be captured, recorded, and transferred to an external PC/Mobile Phone or Cloud for further analysis. Ultimately, the physiological signals intended to be extracted from the raw data are,

1. Breathing pattern (rate/variability)
2. Cardiac cycle (rate/variability)
3. Body movement (body position/activity)

A visualization of the system architecture is shown in Figure 1a and is based on the system requirements.

**Figure 2:**
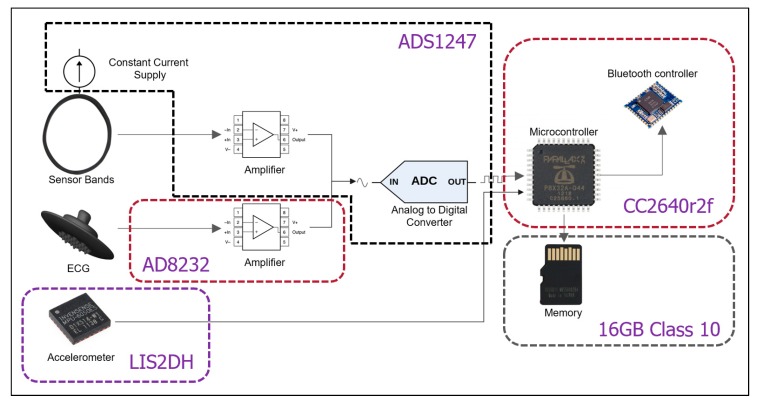
Components inside the T-shirt.

Figure 2 shows each of the components of the T-shirt electronics and the architecture of data flow. The sensor bands are polarized with a constant current supply such that the output voltage changes relative to the resistance changes of the sensor band. The band output and ECG frontend go through two different amplifiers to an ADC. The accelerometer and the ADC connect with the microcontroller unit (MCU) directly through a serial peripheral interface (SPI) bus. The SD card connects with the MCU directly via a high-speed SPI bus.

A 3.7 V, 150 mAh Li-Ion battery measuring 19.75 mm × 26.02 mm × 3.8 mm was chosen to balance the power needs and device footprint size. The total continuous current dissipation of the system must be limited to 15 mA to operate the system for at least 10 h. The minimum sample rate for chest and abdominal measurements from ERBs is 20 Hz [33] and 50 Hz for ECG [34]; however, 100 Hz per channel is used in all experiments across all channels. 

The Bluetooth (System on Chip) SoC, CC2640R2F from Texas Instruments was chosen as the microcontroller unit (MCU) to meet the design requirements. This MCU has an inbuilt Bluetooth stack, removing the need for an external Bluetooth module.

An ADC with a relatively low sampling rate but high precision is required. The ADS1247 from Texas Instruments was selected for several key reasons. The ADC is designed for use in temperature sensor measurements, which is very similar to our use case. It has a 24-bit Delta-Sigma ADC and a current source capable of providing 50 to 1500 µA constant current and an internal programmable gain amplifier with 1–128 variable gain. By using the ADS1247 as our ADC, we can replace three key components in our design with a single discreet integrated circuit (IC).

The AD8232 is a single-channel heart rate monitor IC in a 4 mm × 4 mm package and operates at 170 µA supply current. The LIS2DH triple-axis accelerometer (2 mm × 2 mm) from STMicroelectronics supports up to 16 g acceleration and up to 5.3 kHz variable sampling rate. It consumes 18 µA current at 200 Hz and varies when the rate goes up/down. Both the ECG frontend and accelerometer are ultra-low-power devices contributing a minimum possible load to the central unit.

A MicroSD card was chosen for data storage because of its low cost, high memory density, replacability, and ease of upgrading. A four-layer PCB was manufactured with dimensions of 41 mm × 40 mm (Figure 1(bii)). As the AD1247 allows three differential channels and one channel is allocated to ECG, the board is limited to two ERBs.

Custom-made U-shaped high resistive ERBs were attached on two sides of the board, dividing chest expansion between the two bands. This allows redundancy but also reduces the risk of sensor trapping when the user lays on their side. The PCB is coated with circuit board lacquer to protect from sweat and is concealed under the T-shirt such that the PCB part is placed just below the sternal bone. Hardware placement is critical to comfort. As both male and female human bodies have a small gap under the rib cage, the electronics (the only rigid part of the device) are placed to occupy this space. Even when the user sleeps in a prone position, we hypothesize that there is a minimum chance that the device causes discomfort to the user. Figure 1(a,bi) show the front side of the device and the front view when ERBs are attached.

Connections to disposable ECG electrodes are routed from a header on the PCB. The electronics and ERBs were attached to the internal side of a t-shirt fabric using an “interfacing” [35] technique popular in clothing manufacturing. With interfacing fabric, the device and T-shirt are connected without any woven threads or sewing. When attached, the electronics and sensors are almost invisible (Figure 1(bi)). 

### 2.3. System Benchmarking and Evaluation

The system was tested to evaluate the performance of each component and provide a benchmark for future work. The tests include the average power consumption, ADC test for sampling rate and noise, SD write throughput, and Bluetooth link throughput. In anticipation of overnight sleep studies, where the device may be in operation for 8 h or more, we have tested the device for significantly longer in a laboratory setup. This was done to detect any unexpected interruptions to the data recording. For five days and 18 h, VitalCore continuously acquired data and saved to the SD card. The data were generated from a function generator KEYSIGHT 33210A (Santa Rosa, California, United States) at 1 Hz ± (20 ppm + 3 pHz) sine signal of ±200 mV with 500 mV offset. The two outputs of the function generator are connected to two ERB pads and sampled at 400 Hz, since the original design is to sample two ERBs, the ECG and an accelerometer at 100 Hz with time multiplexing, therefore producing a 400 Hz total sample rate. Sampling the function generator output at 400 Hz makes the test conditions similar to real-world data acquisition. 

The ECG channel was also tested with an ECG simulator that provides 1 mV peak-to-peak ECG output to verify the functionality. A SERT-2009 (Bainuodai, Tianjin, China) portable ECG/EKG simulator was used in the experiment with right arm (RA), left arm (LA), and right leg (RL) nodes connected to corresponding ECG inputs. The AD8232 produced an amplified signal and connects as an input to the ADS1247 ADC. The ADS1247 samples the signal at the same frequency as the other channels (100Hz). All amplification is provided by the AD8232 and the ADS1247 samples the signal with unity gain.

### 2.4. Physiological Experiments

#### 2.4.1. Respiration

The physiological experiments were designed to evaluate the performance of VitalCore against respiration, pulse, and body position during sleeping and light activity. First, VitalCore is compared with a spirometer (BIOPAC TSD107B & DA100C, Biopac, Goleta, CA, United States) for respiratory event detection over 5 min. Simultaneous recordings were captured from the spirometer and VitalCore followed by data alignment to compare respiratory signals from the two devices. It should be noted that the spirometer and VitalCore acquire breathing events in different manners. The spirometer records exhaled pressure only, while VitalCore records the torso expansion/contraction related to both inhaling and exhaling. The peaks of VitalCore readings were aligned with the starting point of the spirometer readings to compensate for the difference. A 0.1 Hz high-pass filter was used to eliminate DC offset in the VitalCore signal. A subsequent cutoff peak detector (30% of peak-to-peak), with a minimum 1-s distance, could find all the inspiratory peaks available in the experiment. The *findpeaks()* function available in MATLAB was used to find the peaks from both signals [36,37]. The instantaneous respiratory rate was calculated using the time difference between peaks for both signals.

#### 2.4.2. Cardiac Activity

ECG functionality is included in the VitalCore to monitor the heart rate and as a comparison for ERB-derived cardiac activity. After verifying the ECG frontend against an ECG simulator, human experiments were conducted using single-use electrodes attached to the body. Three single-use ECG electrodes (RA, LA, and RL) were used, and RA/LA were placed near the left and right ribcage while the RL electrode was placed just above the right hip. The placement of electrodes was kept under the area covered by the T-shirt (Figure 1a). 

In a separate experiment, ERB data were compared to pulse recordings captured with a pressure transducer attached to the index finger (AD Instruments Dunedin, New Zealand) [38]. As the ERB is sensitive to the chest volume changes due to the heartbeat, this experiment was conducted to validate this capability independent of the ECG.

#### 2.4.3. Sleep Test

Sleep tests were conducted while wearing VitalCore for more than 8 h of sleep. VitalCore simultaneously recorded the ERB, accelerometry, and ECG. Accelerometer data allowed for the detection of sleep position and movement artefacts. The accelerometer is programmed for triaxial ±2 g acceleration and collected as an 8-bit signed output. Calculating the respiratory rate is a trivial task when the acquired signal is clean. Due to the battery operation and shorter sensor bands, the VitalCore output signal is not influenced by the power line noise. The primary noise source is body movement, which is a signal artefact that occurs predominantly during sleep position change events. The 0.1 Hz high-pass filter applied eliminates baseline changes in the signal that may occur around these events. One computationally efficient way of counting the respiratory rate is using a high-pass filter followed by a threshold-based peak detector as described above. Threshold-based peak detection works well in short duration, consistent waves. However, when high-frequency movement artefacts are present within a long duration recording such as a sleep dataset, threshold-based peak detection shows a high number of false-positives. To avoid high-frequency movement artefacts, the signal can be passed through a 1 Hz low-pass filter. The maximum average respiration rate is commonly stated as 0.3 Hz, which is equivalent to 20 breaths per minute.

MATLAB features an excellent function to find the local maxima given the minimum threshold and frequency [37]. The function can also return the width of the peak and its prominence. The prominence of a peak measures how much the peak stands out compared to other peaks due to its intrinsic amplitude and its location. We have observed when both bands are combined, the smallest peaks relating to respiration are approximately 3 mV in amplitude. Therefore, the *findpeaks()* function was employed with a minimum time difference between two peaks of 1 s and minimum prominence of 1 mV to identify a peak. The raw output from two bands was manually marked to assess the accuracy of peak detection. Each two-minute window was exported as an image, and the number of false positives and false negatives were recorded. As a standardized practice, the first and last hour of the sleep data were discarded from the 10.5-h experiment. The remaining 8.5 h data produced 255, 2-min plots to be marked, and each respiratory peak is manually marked for comparison.

The respiratory period can be calculated by differentiating the peak position in time and converting to respiration per minute. Respiratory rate variability (RRV) can be presented as the root mean square of the successive differences (RMSSD) of respiratory rates, which is a similar metric that is commonly used in heart rate variability analysis [39]. RR and the corresponding successive differences were computed breath-to-breath for analysis followed by RMSSD calculation using a breath-to-breath RRV array. A healthy respiratory rate for an adult human should be between 12 and 20 breaths per minute, and a respiratory rate higher than 24/min is considered a sign of serious illness [40,41,42,43,44]. Respiratory rate variability could also be presented as a Poincare plot where the x-axis is the n-th respiration rate calculated and the y-axis is the (n + 1)^th^ rate, which are both plotted in breaths per minute (Appendix A.1).

Heart rate is the other measured vital signal from VitalCore. It could be measured and presented as an averaged value over a minute-by-minute basis or beat-to-beat measurement. Measuring continuous beat-to-beat heart rate and heart rate variability could help assess sleep quality, sleep staging, and general health [45,46,47,48,49,50,51]. Since the aim of having an ECG is to gather information about the heart rate and variability, and not acquiring a precise medical-grade ECG waveform, the lead placement could be flexible. During pre-processing, the ECG data were passed through a first-order high-pass filter with a cutoff frequency at 0.3 Hz, and a seventh-order Butterworth low-pass filter with a 15 Hz cutoff value. The filtered data were used with the MATLAB *findpeaks*() function to identify peaks with 150 mV as the prominence value. The dataset chosen was the 9-h sleep data set recorded with VitalCore.

An embedded accelerometer is used to identify movement artefacts and sleep position. The accelerometer returns each x, y, z axis data at 100 Hz as a one byte signed number, which is calibrated to ±2 g at full-scale reading. The following algorithm is used to find regions of high activity.

1. Take the squared sum of each x, y, and z channel for each sample.
  $$SS_{n}=x^{2}+y^{2}+z^{2}$$
2. Calculate the absolute difference between subsequent samples.
  $$\Delta SS=\left|SS_{n}-SS_{n-1}\right|$$
3. Calculate the mean (µ) and standard deviation (σ) of the differentiated array (ΔSS) for 10-s windows. Add mean (µ) to 3σ to cover 99.7% of the values.
4. Apply a 10-s moving mean filter to the µ + 3σ values.
5. Use the *findpeaks*() function to find peaks with a prominence >50.
6. Use peak width at half prominence to mask the high activity regions. The masked region is twice the peak width.

**Figure 3:**
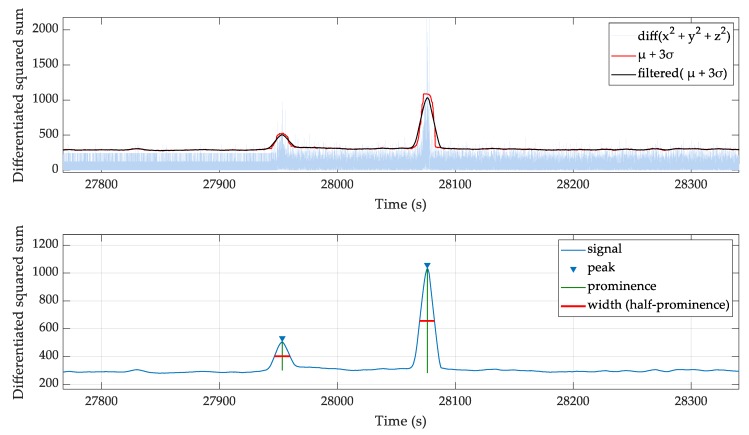
The squared sum of accelerometer channels and the usage of filtered moving standard deviation to identify regions of high activity blocks.

As shown in Figure 3, the squared sum of accelerometer channels and filtered moving standard deviation identifies regions of high activity. The half prominence width is used to generate a high activity masking array.

When calculating sleep position, the accelerometer data requires smoothing as high-frequency oscillations are unrelated to sleep position. This was achieved for all three axes by applying a two-second moving average filter. The x-axis and y-axis were used to calculate angles in radians using an atan2(x,y) operation [52] and converted to degrees by multiplying by 180/π. The percentage of time spent on each sleep position (supine, face-down, left and right) and a circular histogram were chosen to present sleep position data.

#### 2.4.4. Performance during Gait

Finally, to analyze the noise immunity of ERBs when movement artefacts are present, VitalCore was worn during moderate pace walking experiments. The baseline respiratory wave is acquired simultaneously with a stationary, high flow pneumotach spirometer. The two recordings were synchronized by aligning two distinctive respiratory sync patterns performed before and after each experiment. Four data recordings were taken at 1, 2, 3 and 4 km/h walking activity on a treadmill. VitalCore band output was filtered using Singular Spectrum Analysis (SSA) technique [53]. SSA decomposes the time series data into a sum of components. Ideally, VitalCore output would be split into two categories, the respiratory wave and movement artifact. The SSA algorithm was implemented in MATLAB by Javier et al. [54] based on [55]. The parameter L (sliding window length), was chosen as 100 samples and uses the first three components to reconstruct the noise suppressed VitalCore output. The ground truth pneumotach spirometer data were also filtered using the same parameters. The filtered data passed into a prominence based peak detection algorithm to identify each peak related to the maximum inspiration point.

## 3. Results

### 3.1. Hardware and Verification Tests

Table 3 summarizes results from benchmark tests, while Table 4 summarizes the results obtained from long term continuous operation tests.

In the long-term operational test, a total of 499,628 sine wave cycles were produced for recording and VitalCore captured all the sine wave cycles. VitalCore captured 49,962,475 samples per channel at 100 Hz, whereas it was expected to capture 49,962,800 samples (assuming the function generator clock is an accurate baseline). For over five days and 18 h, it missed 325 samples, which was a 0.00065% timing error. For our purposes, considering the relatively slow periodicity of respiration and cardiac activity, this is acceptable. The 1 ms ambiguity in terms of the maximum and minimum sine wave period is a result of the peak “real” sine wave occurring between samples. Vitalcore would record the peak in the next sample or the previous sample resulting in a 0.01 s time resolution error. No interruptions in data capture were noted throughout the experiment. We have confirmed that the ECG frontend produces an accurate waveform using an ECG simulator with a 1 mV input ECG signal.

#### 3.1.1. Physiological Experiment 1: Respiratory Rate and Respiratory Rate Variability

**Figure 4:**
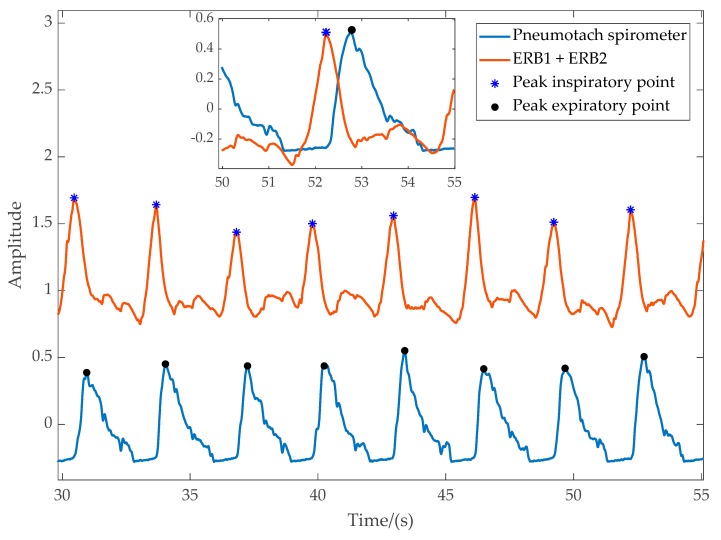
Data captured from the pneumotach spirometer flow and VitalCore. Inset: overlapped data excerpt.

Figure 4 shows readings from the spirometer and VitalCore. Peaks are marked for peak inspiration for VitalCore and peak expiration for spirometer data. The inset highlights the time difference between the two signals. The peak detection and respiratory flow calculation are elementary for VitalCore data due to the clean output it produces. The mean percentage error for the instantaneous respiratory rate compared to the spirometer was only 0.087% breaths/minute with a standard deviation of 3.2%. When averaged over time, the calculated respiratory rate from the spirometer was 19.7191 breaths/minute, while VitalCore shows 19.7179 breaths/minute.

#### 3.1.2. Physiological Experiment 2: ECG Frontend

Human ECG recordings from the Vitalcore ECG frontend showed consistent QRS peaks of approximately 400 to 800 mV amplitude. The recorded wave was passed through a peak detection function with 250 mV prominence cut-off to derive the continuous beat-to-beat heart rate. For the recorded region, 86.48 ± 2.33 mean beats/minute were calculated. The overall quality of the signal was deemed sufficient for the calculation of heart rate and heart rate variability. 

#### 3.1.3. Physiological Experiment 3: ERB-Derived Cardiac Activity

We observed that ERBs are sensitive to the blood volume change due to the heartbeat. The concept is similar to ballistocardiography (BCG); however, we do not measure the force to extract the pulse information. The ERBs measure the tiny volume change of the chest due to the cardiac output and blood flow from the heart. The expansion is apparent in the raw signal when the breathing artefact is removed.

**Figure 5:**
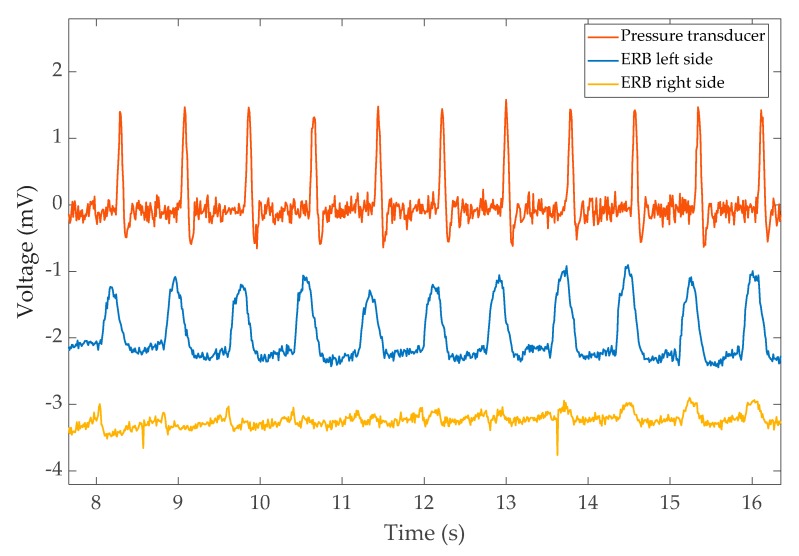
The pulse recorded from the pressure transducer and the corresponding reading from left/right bands of VitalCore. The readings were taken while holding breath.

The output from the finger pressure transducer along with the left-side and right-side ERBs are shown in Figure 5. Interestingly, the left band shows the expansion due to cardiac activity much more clearly. A time difference between the peaks of finger measurement (PPG) and heartbeat expansion from the ERBs is also visible. The time difference corresponds to the pulse transient time. 

Measuring the heart rate from the left band is a trivial task if the respiratory movements are not involved. However, when respiratory movements are involved, the task requires further post-processing. The heartbeat is visible to the naked eye even when respiratory movements are present in the captured waveform; however, getting a consistent automated measurement requires further analysis of the signal.

**Figure 6:**
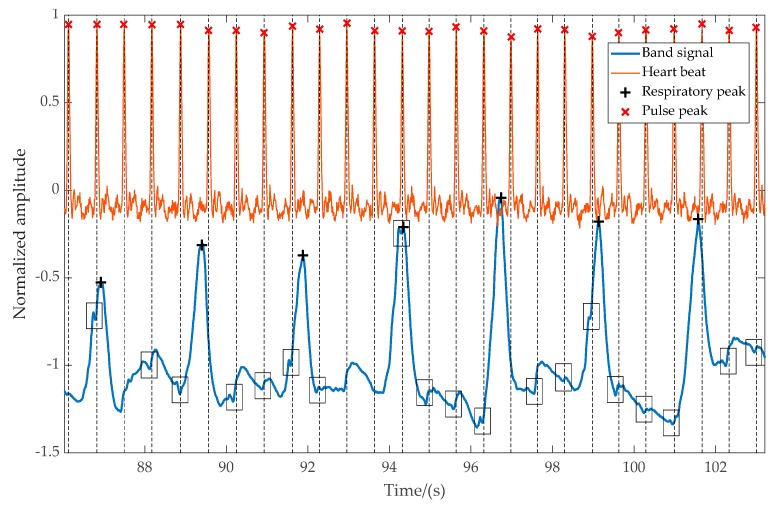
The magnitude of the respiration signal and pulse signal from the left-side band compared to heartbeat captured from a finger-worn pulse transducer. The heartbeat from the band is visible as small negative glitches, which represent the starting point of the pulse.

Figure 6 shows an excerpt of captured data recorded with a pressure transducer during regular breathing. The output of the pressure transducer and detected peaks are projected downwards to the overlayed VitalCore measurements. The larger peaks are respirations, while smaller glitches indicated in black boxes are the sudden variation of blood volume. It is evident that even though the heartbeats are clearly visible, false-positive rejection and a higher degree of filtering is required to extract the heartbeat only from VitalCore bands. 

### 3.2. Sleep Monitoring

Sleep data have some unique features. The artefacts are comparatively small compared to those during activity (e.g., walking) unless the test subject is suffering from significant sleep disturbance. Most of the artefacts come from voluntary or involuntary movements such as those that occur due to changing sleep position and arousals. 

#### 3.2.1. Full Night Recording

**Figure 7:**
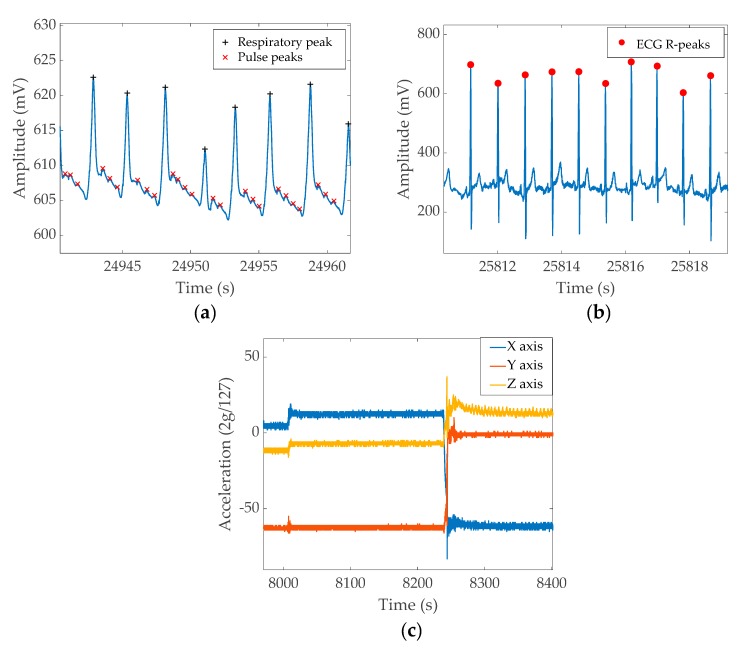
Example signal patterns from a full night recording. (**a**) ERB output, (**b**) ECG output, (**c**) X, Y, and Z axis output from the accelerometer. The figures show different sections extracted from the full night recordings.

Figure 7 shows the sample output from each channel recorded during sleep.

Figure 7a shows the unfiltered ERB output. The signal has a significantly large DC offset while the signal corresponding to respiratory function results in 10–20 mV change. The smaller peaks between respiratory events are heartbeats. Figure 7b shows the unfiltered ECG recordings. The QRS peak is about 100–200 mV on average; however, it could increase to 400 mV on some occasions. The visible oscillation of the ECG peak value is due to the respiratory movement. Figure 7c shows the output of the three-axis accelerometer. The chosen section demonstrates three essential pieces of information that could be acquired from the accelerometer; i.e., the first half of the figure shows a relatively still position and a small movement occurred around the 8000 s mark. Then, around 8250 s, the subject changes sleep position, resulting in a more significant artefact. A significant wave pattern in the Z axis at the end of the graph is the respiratory output captured as the acceleration change. The data windows for both the band signal and ECG wave were chosen from near the end of the full night data recording, demonstrating that the band/ECG signal maintains signal quality throughout the full recording. 

#### 3.2.2. Respiratory Rate and Variability 

Respiratory rate and variability measures could be performed quickly using automated functions. The experiment data used to find the respiratory rate and variability had 10.5 h of raw data and 8.5 h of useful region. The *findpeaks()* function returned peaks, timestamps, peak height, and width based on prominence. A total of 6498 peaks were extracted, 245 peaks were identified as false-positives, and 35 peaks were missed (i.e., false-negatives). This represents a sensitivity of 99.44%, a precision of 96.23%, and a false-negative rate of 0.557% in identifying respiration peaks. The algorithm took only 0.55 s using an Intel I7 6820HQ CPU with 16 GB memory. The 6498 identified peaks have a mean respiration rate of 14.52 respirations/minute with a standard deviation of 7.23. The majority of the data (89% of total respirations) lie below 20 breaths per minute. The root mean square of the successive differences (RMSSD) value for respiratory rate variability was computed as 7.06 breaths per minute. 

**Figure A1:**
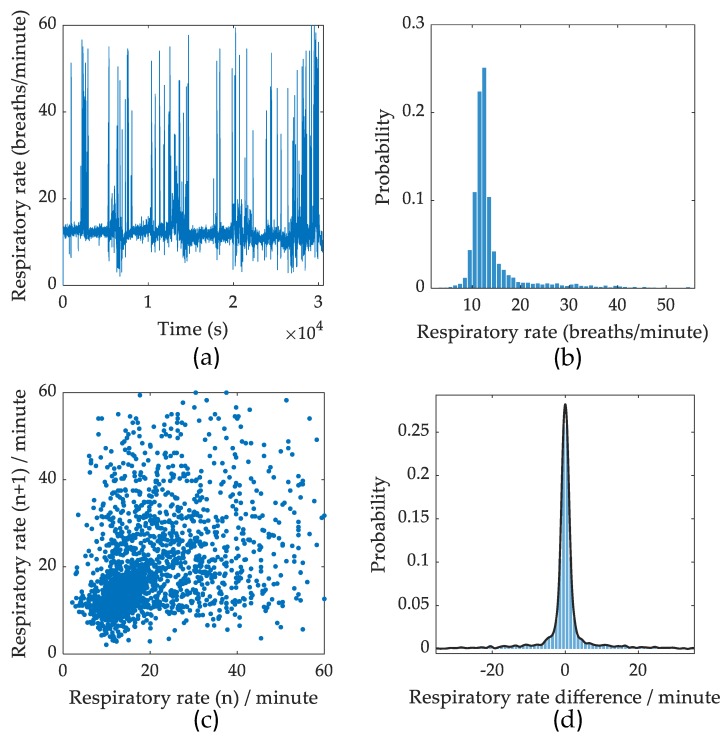
Respiratory rate and variability representations. (**a**) Respiratory rate. (**b**) Distribution of respiratory rate. (**c**) Poincare plot of respiratory rate variability. (**d**) Probability distribution of respiratory rate variability.

Appendix A, Figure A1 shows the calculated breath-to-breath respiratory rate, a histogram summary of the respiratory rates, a Poincare plot showing the distribution of successive respiratory rates, and a histogram of the respiratory rate variability (RMSSD) calculated for each respiratory event. 

#### 3.2.3. Heart Rate and Heart Rate Variability 

QRS peaks from VitalCore varied between 200 and 300 mV generally. A 9-h sleep dataset produced 36,173 ECG QRS peaks. Processing to identify peaks took 1.5 s, and the filtering stage took 0.7 s. The resulted mean heart rate was 68.82 beats/minute with a standard deviation of 14.59 beats/minute. The identified R-R peaks were used to calculate heart rate variability. We observed a sudden rise of heart rate correlated with sleep position changes as verified against the accelerometer data. When the subject changes position, the ECG output becomes unstable; however, it immediately stabilizes after the subject lies still. The majority of heart rate values lie around 55–85 beats/minute. The recorded dataset shows a high heart rate variability RMSSD value of 13.67 beats/minute change, indicating a healthy subject. 

**Figure A2:**
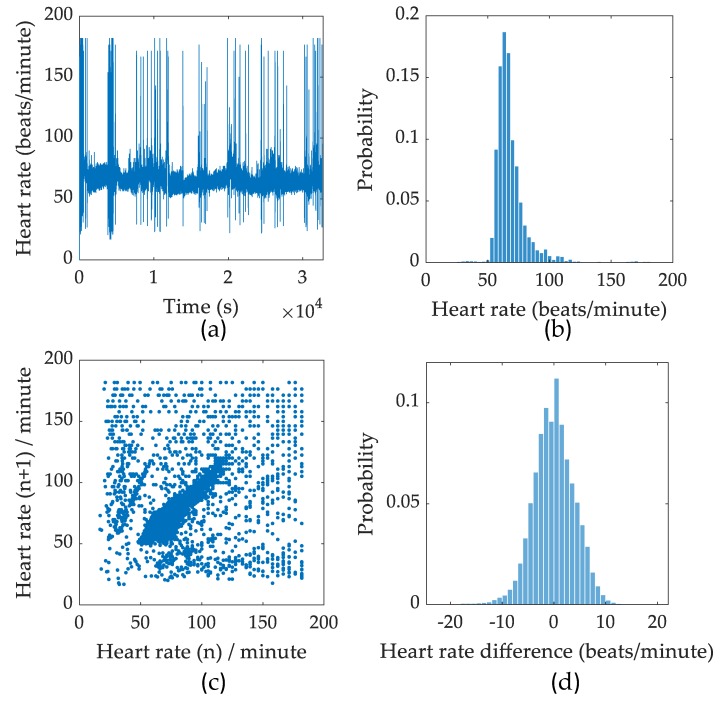
Summary of analysed ECG recordings. (**a**) Beat-to-beat heart rate variation. (**b**) Histogram of heart rate and corresponding probability. (**c**) Poincare plot of n^th^ heartbeat against (n + 1)^th^ heartbeat. (**d**) Histogram of heart rate difference.

The ECG data are shown in Appendix A, Figure A2 and includes calculated beat-to-beat heart rate, a histogram summary of the heart rate, a Poincare plot showing the distribution of successive beat-to-beat heart rate values, and a histogram of heart rate variability (RMSSD) calculated for each pulse event captured. 

#### 3.2.4. Actigraphy and Movement Artefact Rejection 

**Figure 8:**
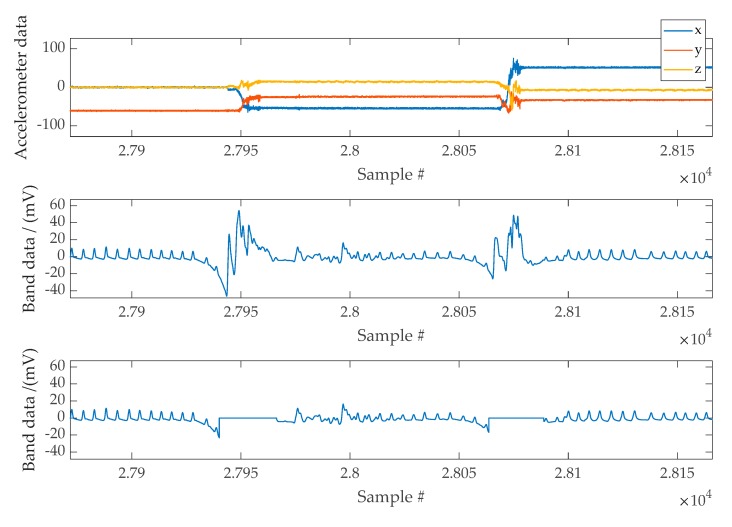
Top Panel: Example of accelerometer data used to mask high activity regions. Middle Panel: Band data affected by moving artefacts. Bottom Panel: Band data following the masking of movement artefacts.

The accelerometer is a versatile device that gives valuable sleep information when adequately placed. Since VitalCore hardware was placed on top of the chest, the angle between gravitational acceleration and VitalCore is directly related to sleep position. In addition, the accelerometer provides information about motion artefacts that help reject the band data within this period. When used for artefact removal, the squared sum of all three axes could be considered as a single input regardless of the acceleration direction. Figure 8 shows a section of cleaned band data when a high activity mask is used to remove moving artefacts.

#### 3.2.5. Sleep Position and the Effect of Respiratory Output 

Having the accelerometer on the chest gives a clear advantage when the sleep position needs to be calculated. As VitalCore hardware was positioned on top of the chest and the subject sleeps horizontally, sleep position can be calculated by calculating the roll angle. 

**Figure 9:**
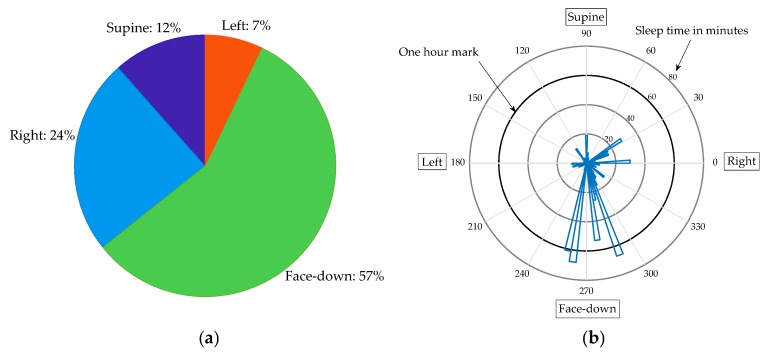
Summary of sleep position from a full night recording. (**a**) Percentage of time spent in each position. (**b**) A polar diagram showing sleep angle versus sleep time in minutes.

Figure 9 summarizes a full night sleep recording. The first plot shows the percentage of time spent on the nearest estimation to each sleep position. The second polar diagram shows the time spent at each angle (binned to 4 degrees).

The sleep position calculation shows an important advantage of VitalCore. As observed from Figure 9, this user spent the majority of time sleeping face-down, which is presumably the most comfortable sleep position for the subject. This is not possible in polysomnography tests and difficult to achieve using current at-home sleep apnea test devices. 

The ability to operate in every position is useless if the device cannot provide a reasonable measurement from each position. The artefact-removed signal was passed through a prominence-based peak detection algorithm that returns the peak height for each peak identified. The peak height vector is multiplied by the sleep position mask generated for each sleep position to obtain the peak heights in each position. Table 5 shows the statistics for peak heights based on each position. The trimmed mean was measured by removing 5% of data outliers from each side of the data.

As shown in Table 5, there is a negligible difference in the mean height between the supine and face-down position. The right-side position shows a strong signal with low standard deviation followed by the left-side position. However, the left side signal has a high standard deviation, with the mean value being slightly higher than the face-down position.

**Figure 10:**
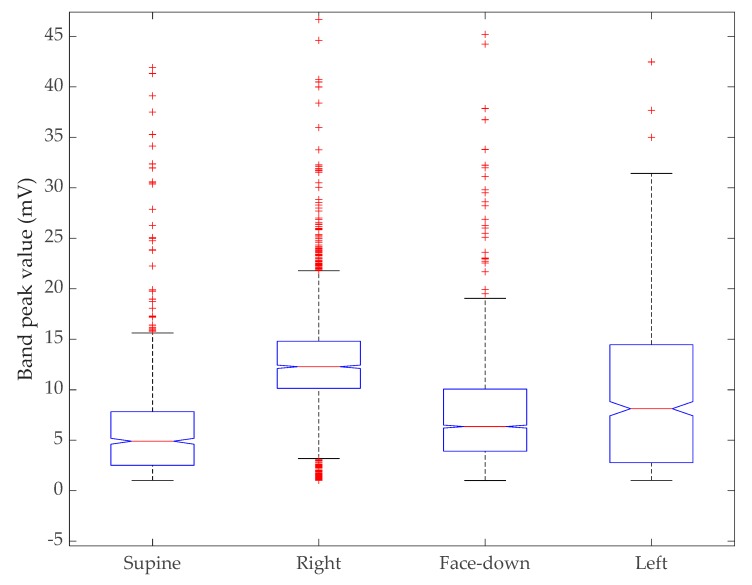
Results of Kruskal–Wallis test. The box plot shows the distribution of peak values of the sensor band versus sleep position.

We conducted a Kruskal–Wallis test to compare all four sleep positions together. Figure 10 shows the distribution of the peak values in a box plot without reducing the sample size for each group. The Kruskal–Wallis test is a nonparametric version of one-way ANOVA and an extension of the Wilcoxon rank sum test to more than two groups. This test compares the medians of the groups of data to determine if the samples come from the same population (or equivalently from different populations with the same distribution). With a p-value of zero, the test rejects the hypothesis that all samples come from the same distribution. Table 6 shows the resulting ANOVA table using standard notation.

Further, we compared pairwise groups using the Wilcoxon rank sum test [56] after producing a probability density estimation for all sleep positions. The rank sum function tests the null hypothesis that data in two distributions (x and y) are samples from continuous distributions with equal medians, against the alternative that they are not. All four positions were compared pair wise and it was noted if two positions could reject the null hypothesis at the 5% significance level or if they failed to do so. All combinations except supine versus left and face-down versus right reject the null hypothesis, as shown in Table 7.

Another characteristic measure is how differently each band behaves when the user changes position. VitalCore is designed such that the device is centered while two bands extend to the left and the right so that even if one side is trapped, the other band could still function. For example, if the user is sleeping on their left side, the left band might be bearing bodyweight, resulting in a low response to physiological activity; however, as the right-side band is free from load, we hypothesize that it still can capture respiration. As the accelerometer captures a sleep position mask for each side, we tested this hypothesis by measuring the signal power acquired from each position. The signal power is considered as the root mean square for both bands for each position independently. 

Table 8 confirms the hypothesis that VitalCore bands can complement each other when measuring sleep data. The supine and face-down positions show a small difference between the two bands while the left and right positions show a considerable difference. Clearly, when in the left position, the right band is free to move and provides a better signal, while in the right position, the left band provides a better signal.

#### 3.2.6. Respiratory Rate Calculation Using Accelerometer Readings 

Apart from using the accelerometer to reject movement artefacts and calculate the sleep position, we observed that the accelerometer could show respiratory data. It is possible to remove high-frequency noise from the accelerometer and obtain a reasonably comparable respiratory rate calculation. As the X and Y axis are used to estimate the sleep position, the Z axis of the accelerometer records the respiratory data. However, when the subject sleeps on their side, it is observed that X axis of the accelerometer also provides the respiration signal. Therefore, the squared sum of the X and Z axes was used to analyze the respiratory pattern from the accelerometer. A 20th-order Butterworth filter with a stopband gain of 80 dB and passband frequencies of 0.66 Hz and 1 Hz was created to filter the accelerometer data. Sleep position masks for supine and face-down were used to invert the accelerometer readings as the peak inspiration point of face-down is opposite to that of the supine recordings.

**Figure 11:**
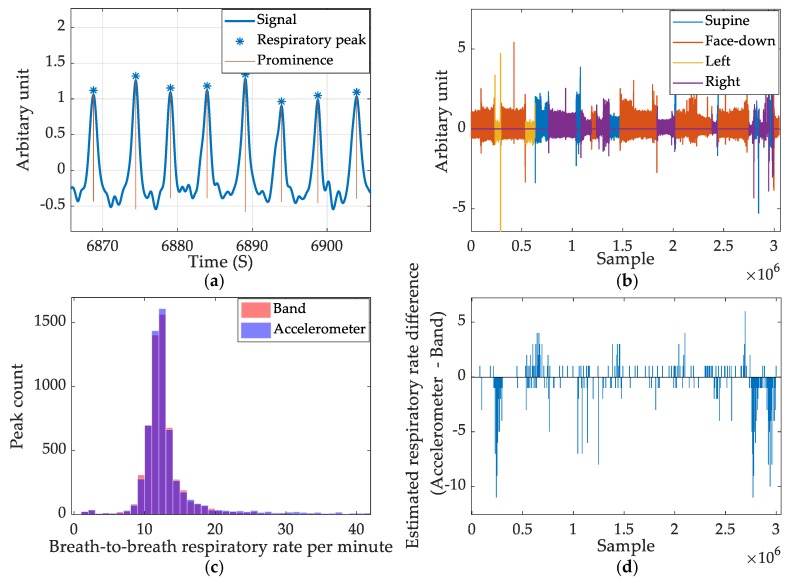
Summary of accelerometer-based respiratory rate estimation. (**a**) The respiratory signal captured by accelerometer and detected peaks. (**b**) The color-coded respiratory signal captured in a full night’s recording. (**c**) Breath-to-breath respiratory rate count comparison between accelerometer and band signal. (**d**) The estimated respiratory rate difference between the accelerometer and band data.

The accelerometer-based respiratory measures and band-produced respiratory measures are compared graphically to summarize the comparison of respiratory peak detection between band data and accelerometer data. Figure 11a shows a section of respiratory wave filtered from the accelerometer and detected peaks from the ERBs using the prominence-based approach. Figure 11b shows the color-coded accelerometer-derived respiratory data based on sleep position. The left/right positions yield the weakest signal amplitude. Figure 11c shows the histogram of both the band derived respiratory rate and accelerometer-derived respiratory rate. The two colors, red and blue, combine to purple where both parameters yield identical outputs. Figure 11d shows the error calculated using the ERB-derived respiratory rate as the ground-truth for accelerometer measurement. It is aligned with Figure 11b to show that the highest error occurs when the subject sleeps in side positions.

#### 3.2.7. Respiration Rate Calculation during Light Activity

VitalCore is not designed as an activity monitor nor as a sports monitor. In sleep monitoring applications, the subject is relatively static compared with day-to-day activity or during exercise. However, we observed that VitalCore could be used to monitor breath-to-breath respiration during light activities. The majority of current activity monitors utilize accelerometry, pulse oximetry, and GPS where the main focus is to track heart rate during activity. Most of the devices lack the capability of measuring respiration directly. VitalCore has some advantage in this regard, as it can measure respiration directly instead of relying on indirect inference. The fixed position of the sensors on the chest (in comparison to wrist-worn devices) makes the accelerometer pattern for activity highly predictable and repeatable, resulting in simpler data processing.

**Figure 12:**
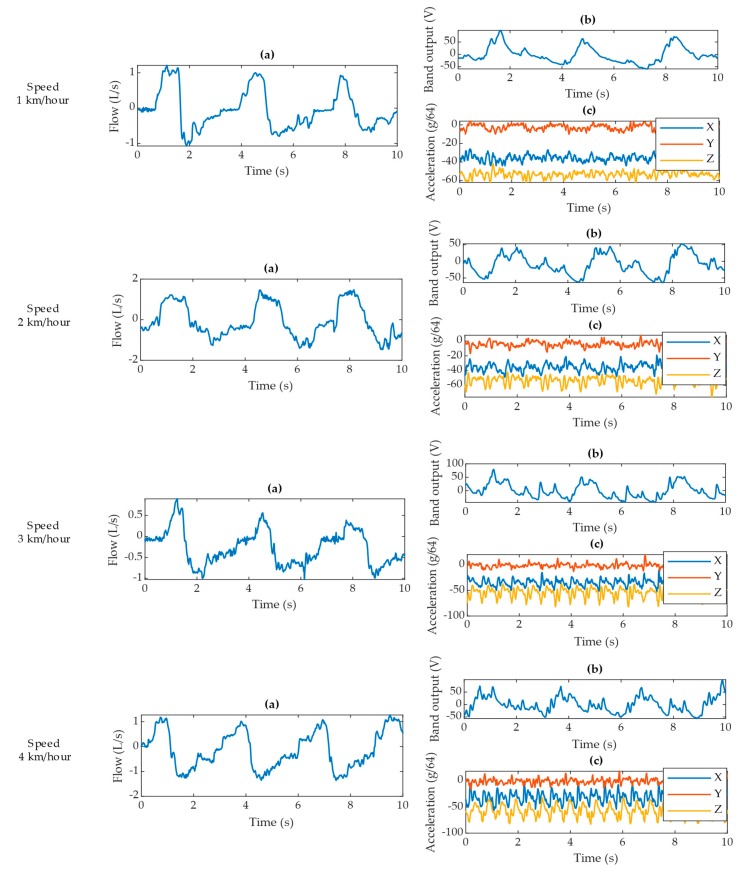
Data excerpts for each walking speed showing (**a**) pneumotach spirometer output, (**b**) corresponding VitalCore output from two bands, and (**c**) corresponding accelerometer readings from VitalCore.

During gait at four different speeds, the pneumotach spirometer produces a consistent signal for all four scenarios (Figure 12). The VitalCore respiratory response degrades with walking speed and the accelerometer starts to produce a prominent pattern for walking activity.

**Figure A3:**
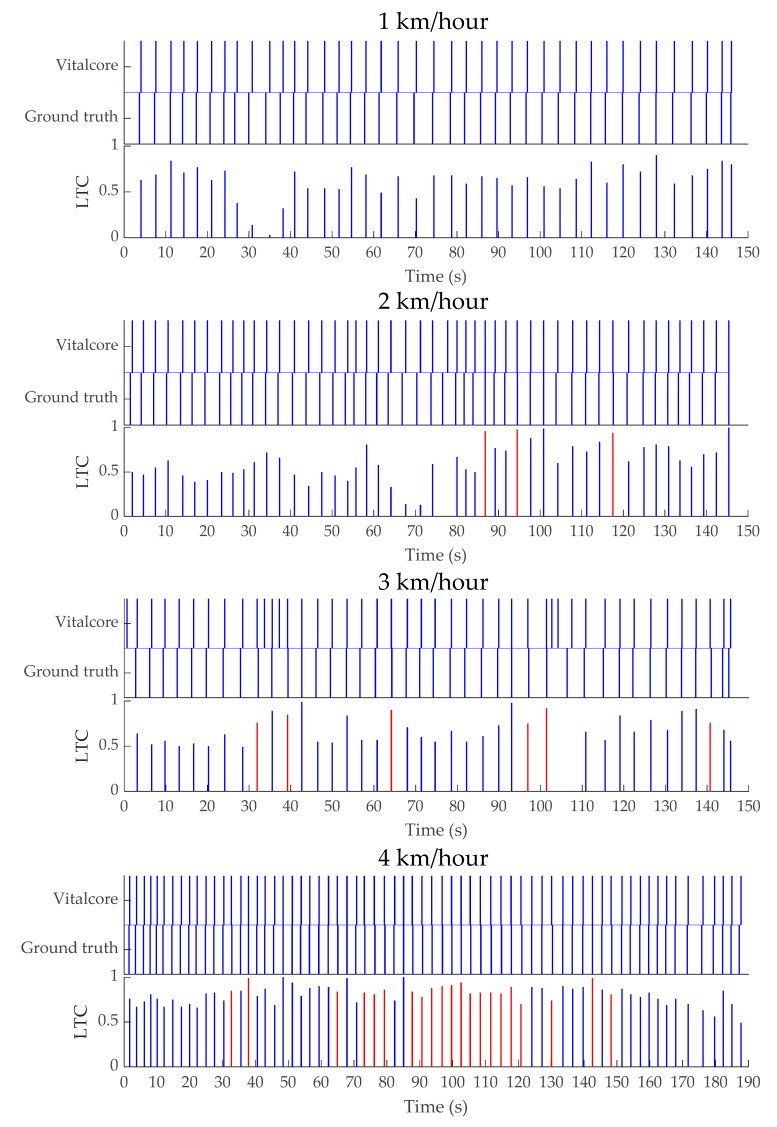
Timestamp graph for ground truth and VitalCore with linear temporal correlation (LTC) between ground truth and VitalCore for walking speeds of 1 km/h, 2 km/h, 3 km/h, and 4 km/h.

We present the linear temporal correlation (LTC) for the respiratory peaks detected from ground truth data and VitalCore data in Table 9. Surprisingly, the highest correlated and highest accuracy respiratory event detection is achieved when walking at higher speed (4 km/h), as shown in Table 10 and Figure A3. The 4 km/h VitalCore dataset presents no false-positives and shows the highest correlation between the corresponding peaks. This could be due to the faster walking introducing high-frequency movement artefacts that are significantly different from the respiratory frequency, so it is easier to remove these artefacts from VitalCore data. Table 10 summarizes all four walking speeds in terms of the mean respiratory rate, standard deviation, and step frequency derived from Fast Fourier Transformation (FFT). The data from Table 10 do not conclusively prove that VitalCore can detect all respiratory events during light activity due to the low experimental sample size. However, the experiments show that VitalCore ERBs and relatively simple algorithms are quite tolerant of movement artefacts. 

## 4. Discussion

This paper presents the evaluation of VitalCore, a truly wearable device designed for vital signal monitoring, specifically targeting low activity and sleep monitoring applications. The experiments were conducted in a laboratory setup and home environment. Initial experiments evaluated device reliability during long-term bench testing, examining Bluetooth function and data writing to confirm that the device is capable of running uninterrupted for 8 h or more. Furthermore, the device was tested with a portable ECG function generator and cross-compared with a pneumotach spirometer and finger pulse transducer to verify the expected functionality. Then, the device was evaluated in full night sleep studies capturing data continuously for more than 8 h.

The signal output of the bands proved to be clean with high SNR, allowing simplified data-processing algorithms. The processing time for identifying respiratory peaks for a 10.5-h dataset took 0.55 s and resulted in 99.44% sensitivity, 96.23% precision, and a 0.557% false-negative rate for respiratory peak detection. The ECG frontend worked reliably and showed minimum obstruction and peak-to-peak difference. The processing of ECG took 2.2 s, including the filtering stage.

The most versatile sensor of the system was the accelerometer. While of little use on its own, when combined with bands and ECG, it resulted in valuable insight into sleep patterns, and it could be used as a reliable sleep position estimator due to the center chest placement. The accelerometer could also be used to highlight high-activity regions from the dataset and highlight these for sleep analysis or artefact rejection. Accelerometer readings were sensitive enough for respiratory monitoring but not suitable for all sleep positions.

The positioning of two bands on opposite sides of the electronics allowed them to complement each other. When sleeping on either side, the mean signal amplitude was reduced. However, the unloaded band has a high SNR compared to the band bearing body weight. This supports having two independent bands on each side of the body in sleep setups. Moreover, we found the left band is more sensitive to the heartbeat compared to the right-side band. 

As each band is polarized with 100 µA current sources, the sensor interface introduces very low-power consumption toward the total power budget. Since the resistive measurement can be conducted with a single integrated ADC package (ADS1247) and no filtering or post-processing of the signal is required, VitalCore needs fewer and simpler discreet components compared to other devices available in the market. It allows us to produce a smaller PCB, which leads to comfortable wearability due to the small size of the device. The bands are highly sensitive to body volume changes and sufficiently sensitive to allow heart rate estimation using the band data only. This could lead to electrodeless heart rate estimation in the future, but it will require the development of bespoke post-processing approaches in the future.

These resistive elastomer sensors are prone to capturing body movement when placed on the chest. As VitalCore band sensors and accelerometer are in proximity, there is the possibility of distinguishing the source of the activity (e.g., torso/non-torso). The U-shape of these bands allows completion of the electrical loop via the sensor material, so no additional wiring is required. This reduces the complexity and potential points of failure. Their stretchability allows capturing the respiration and heartbeat without applying significant force to the wearer, while their flexibility allows the sensor to wrap around the chest, extending the sensor length and increasing sensitivity. Further, the U-shaped sensor design minimizes the risk of trapping when the user sleeps on their side positions. The base sensor material is low cost and readily available in the market as electromagnetic interference gaskets. The material may be custom molded into a variety of shapes allowing a greater flexibility of sensor design. Finally, VitalCore allows best in the class full-speed Bluetooth 5 connectivity with Bluetooth 4.2/4.1 backward compatibility. Up to 32 GB swappable microSD storage enables at least 200 days of data recording in continuous operation (at 1.6 kBps).

However, VitalCore hardware is not without its limitations. We believe that the current 41 mm × 40 mm PCB size could be further reduced using smaller discrete devices and using a denser component placement. VitalCore morphic band sensors do capture non-torso-related movement, in particular arm movement. Further work is required to either eliminate this artifact through improved physical design or by using accelerometer data to filter out this unwanted signal. The most critical disadvantage of VitalCore is the lack of blood oxygen saturation (SPO2) sensor. SPO2 is widely used in sleep monitoring; indeed, the definition of sleep apnea is defined using SPO2 [57]. With our current design, obstructive breathing events would have to be inferred using the respiratory wave shape, wave height, and associated accelerometer readings. Dedicated sleep studies comparing to gold standard polysomnography will determine if this is feasible. The alternative is to include an SPO2 sensor; however, to do this without affecting user comfort is a significant challenge in itself.

This work focuses on the evaluation of a proof-of-concept version of VitalCore and is limited due to the small dataset produced. Future work will evaluate the use of VitalCore in a heterogeneous cohort of subjects with varying body shapes and those with cardiovascular and/or respiratory conditions with a primary focus on sleep disorders.

Finally, VitalCore is designed with user comfort as a priority. The designed hardware induces minimum to no obstruction to a comfortable sleep. The test subjects showed no sign of discomfort and did not wake up during sleep due to discomfort. Importantly, VitalCore allows sleeping in any sleep position, as shown in the results. The morphic bands still managed to capture respiratory function at high accuracy in all positions. This further solidifies the utility of electroresistive materials in vital monitoring applications.

## Figures and Tables

**Table 1:** Summary of the current state-of-the-art vital monitoring wearables and internal technology. ECG: electrocardiogram, HR: heart rate, PPG: peaks of finger measurement, RR: respiratory rate.

Product	Purpose	HR	HR Technology	RR	RR Technology
Everion [14]	Fitness/medical	✓	PPG	✓	—
Hexoskin [15]	Fitness/medical	✓	ECG	✓	RIP bands
Ambiotex [16]	Fitness	✓	ECG	✗	—
Athos [17]	Fitness	✓	ECG	✗	—
Hitoe [18]	Fitness/consumer	✓	ECG	✗	—
Roudjane et al. (2018) [19]	Consumer	✗	—	✓	Wireless antenna signal attenuation
Presti et al. (2019) [20]	Sports	✗	—	✓	Flexible Fiber Bragg Grating
Trindade et al. (2016) [21]	Consumer	✓	ECG	✗	—
“Phyjama”, Kiaghadi, A. (2019) [22]	Consumer/medical	✓	Resistive sensor fabric	✓	Resistive sensor fabric
Our work	Consumer/Medical	✓	ECG + Carbon black elastomer	✓	Carbon black elastomer

**Table 2:** Electrical and physical characteristics of a U-shaped rubber band.

Parameter	Value
Length	11.6 mm
Width	2 mm
Thickness	2 mm
Resistance/cm	258.6 Ω
Δ Resistance/cm	500 Ω

**Table 3:** System benchmarks.

Parameter	Value	Comment
Power consumption	12.28 mA	Average current used by all the peripherals
Signal-to-noise ratio	84 dB	DC signal
SD data write	375 kB/s	Maximum
SD data read	538 kB/s	Maximum
Bluetooth link throughput	1300 kb/s	Using Bluetooth 5

**Table 4:** Summary of observed parameters from long-term continuous operation test and expected values.

Parameter	Observed	Ideal Value	Error
Input sine waves (n)	499,628	499,628	0
Time for n waves	499,624 s	499,628 s	0.000650%
Sampling rate	399.9968 Hz	400	0.0032 Hz
Maximum period	1.01 s	1 s	1 ms
Minimum period	0.99 s	1 s	1 ms

**Table 5:** Descriptive statistics for respiration peak heights for each sleep position. Peak height data measured in mV.

Sleep Position	Mean	Trimmed Mean	Standard Deviation
Supine	6.07	5.32	5.71
Left	9.33	8.59	8.12
Right	13.03	12.59	6.68
Face-down	7.16	6.91	4.32

**Table 6:** Kruskal–Wallis ANOVA table for sleep position comparison.

Source	SS/(10^10^)	Degrees of Freedom (Df)	Mean Squares (MS)	Chi-sq	Prob > Chi-sq
Groups	0.830654	3	2.7688 × 10^9^	1786.09	0
Error	2.64295	7466	3.5399 × 10^6^		
Total	3.47361	7469			

**Table 7:** Pair-wise *p* value from Wilcoxon rank sum test.

Sleep Position	Left	Right	Face-Down
Supine	0.9542	0.00039	0.00024
Left	-	0.00022	0.00014
Right	-	-	0.6752

**Table 8:** Calculated power for each band for each sleep position and the overall power difference.

Sleep Position	Left Band Power	Right Band Power	Power Difference
Supine	0.365	0.300	0.065
Left	0.289	0.535	−0.246
Right	1.890	1.132	0.757
Face-down	1.796	1.704	0.092

**Table 9:** Data statistics for linear temporal correlation (LTC) between ground truth data and VitalCore data for respiratory peak detection.

Walking Speed	Min LTC	Max LTC	LTC Range	LTC Mean	LTC Median	LTC Mode	LTC Standard Deviation
1 km/h	0.24	0.75	0.51	0.4579	0.475	0.49	0.1128
2 km/h	0.13	1.00	0.87	0.6198	0.605	0.50	0.2043
3 km/h	0.49	0.99	0.50	0.6897	0.660	0.55	0.1490
4 km/h	0.49	1.00	0.51	0.8090	0.820	0.83	0.1033

**Table 10:** Mean respiratory rate, standard deviation comparison for VitalCore with walking step frequency, and respiratory peak accuracy.

Walking Speed	Dataset	Respiratory Rate (Breaths/Minute)	Standard Deviation	Step Frequency (Steps/Minute)	Peak Detection Accuracy
1 km/h	Ground truth	16.32	2.51	64.38	100%
	VitalCore	16.53	1.90		
2 km/h	Ground truth	20.42	3.14	83.04	100%
	VitalCore	20.53	3.28		
3 km/h	Ground truth	17.00	4.23	104.40	88.89%
	VitalCore	19.99	7.75		
4 km/h	Ground truth	21.70	3.02	114.78	100%
	VitalCore	21.71	3.13		

## Appendix A

### Appendix A.1. Respiratory Rate (RR), Respiratory rate Variability (RRV), Heart Rate (HR), and Heart Rate Variability (HRV)

A detailed snapshot of a full night recording for RR and RRV measures is shown in Figure A1. Figure A1a shows the RR calculated using the period of successive respiratory peaks. The sudden increase of RR is due to movement artefacts. In practice, these events are observed as single or multiple high frequency oscillations around the time of body movement. Figure A1b shows a summary of respiratory rates. About half of the respiratory rates are within 10-12 respirations/minute. Figure A1c shows a Poincare plot of successive respiratory rates. The x-axis shows the n^th^ RR calculation against the successive (n + 1)^th ^ RR. Poincare plot could be used to visualize the RRV where a distributed graph shows a high RRV while a grouped point in the graph show a low RRV. Figure A1d summarizes RRV. The difference of successive respiratory rates grouped as a histogram.

Similarly, the heart rate (HR) and heart rate variability (HRV) results from a full night recording are shown in Figure A2. Since the HR is generally higher than the RR, the Poincare plot is denser and show a higher HRV value compared to RRV. In addition, the HRV shows a lower kurtosis compared to the RRV.

### Appendix A.2. Respiratory Rate Calculation during Light Walking and Linear Temporal Correlation (LTC) with Ground Truth Data

The respiratory rate calculated during walking speeds of 1 km/h, 2 km/h, 3 km/h, and 4 km/h is shown in a timestamp graph with linear temporal correlation (LTC) measures (Figure A3). The timestamp graph for each peak provides an abstract view of how well each peak from ground truth relates to the corresponding VitalCore-based inspiratory peaks. Perfectly synced peaks would show a straight column, while lag/lead shows as a misalignment. The LTC graph indicates the time difference between VitalCore and ground truth peaks as a value. For each ground truth peak (P_n_ = 1), a linearly decaying function F(t) = P_n_ − 0.01 ∆t where F(t) > = 0, multiplied with corresponding VitalCore peak (V_n_ = 1). Perfectly aligned peaks produce 1 as the maximum correlation while any peak occurring outside one-second lag/lead would produce a value of zero. The LTC is color-coded where blue bars present lagging VitalCore peaks and red bars present leading VitalCore peaks.

A consistent and higher LTC graph shows a high confidence in respiratory peak detection. The 4 km/h test produces a much better LTC graph compared to other walk tests.
